# Investigating coping and stigma in people living with HIV through narrative medicine in the Italian multicentre non-interventional study DIAMANTE

**DOI:** 10.1038/s41598-023-44768-2

**Published:** 2023-10-17

**Authors:** A. Antinori, A. Vergori, D. Ripamonti, D. Valenti, V. Esposito, M. A. Carleo, S. Rusconi, A. Cascio, E. Manzillo, M. Andreoni, G. Orofino, A. Cappuccio, L. Reale, M. G. Marini, D. Mancusi, R. Termini, A. Uglietti, M. Portaro

**Affiliations:** 1 HIV/AIDS Unit, National Institute for Infectious Diseases “Lazzaro Spallanzani” IRCCS, Rome, Italy; 2 Infectious Diseases Clinic, Papa Giovanni XXIII Hospital, Bergamo, Italy; 3 General Infectious Diseases Unit, Department of Infectious Disease and Infectious Emergencies, Cotugno Hospital, Naples, Italy; 4 DIBIC Luigi Sacco, University of Milan, Milan, Italy; 5 Infectious Diseases Unit, Legnano Hospital ASST Ovest Milanese, Legnano, Italy; 6 Infectious Diseases Clinic, AOU Policlinico “P.Giaccone”, Palermo, Italy; 7 Infectious Disease and Infectious Emergencies, Azienda Ospedaliera dei Colli, Naples, Italy; 8 Infectious Diseases Clinic, Foundation Policlinico Tor Vergata University Hospital, Rome, Italy; 9 Amedeo di Savoia Hospital Unit of Infectious Diseases Torino, Turin, Italy; 10 Healthcare Area, ISTUD Srl, Milan, Italy; 11 Medical Affairs Department, Infectious Diseases and Vaccines, Janssen-Cilag SpA, Via Michelangelo Buonarroti, 23, 20093 Cologno Monzese, MI Italy

**Keywords:** Virology, Diseases

## Abstract

Antiretroviral therapy (ART) significantly reduced Human Immunodeficiency Virus (HIV) morbidity and mortality; nevertheless, stigma still characterises the living with this condition. This study explored patients’ coping experience by integrating narrative medicine (NM) in a non-interventional clinical trial. From June 2018 to September 2020 the study involved 18 centres across Italy; enrolled patients were both D/C/F/TAF naïve and previously ART-treated. Narratives were collected at enrolment (V1) and last visit (V4) and then independently analysed by three NM specialist researchers through content analysis. One-hundred and fourteen patients completed both V1 and V4 narratives. Supportive relationships with clinicians and undetectable viral load facilitated coping. Conversely, lack of disclosure of HIV-positive status, HIV metaphors, and unwillingness to narrate the life before the diagnosis indicated internalised stigma. This is the first non-interventional study to include narratives as patient reported outcomes (PROs). Improving HIV awareness and reducing the sense of guilt experienced by patients helps to overcome stigma and foster coping.

## Introduction

The World Health Organisation (WHO) recommends antiretroviral therapy (ART) for all people living with Human Immunodeficiency Virus (HIV), regardless of CD4+ count or clinical stage^1^. This “Treat All” policy is based on evidence that early HIV treatment is associated with better outcomes across the care continuum and reduced HIV transmission^2^. The implementation of the “Treat All” policies recently contributed to increased access to ART; consequently, HIV-related morbidity and mortality declined, and people living with HIV (PLWH) life expectancy increased^3,4^ and, in many settings, HIV treatment shifted to a chronic care model of disease management^5–7^.

A better understanding of the factors influencing health-related quality of life (HRQoL) among PLWH is critical to identify effective strategies to improve their health and well-being; furthermore, improving HRQoL of PLWH has been recognised as a therapeutic aim by HIV treatment guidelines^8^. Although definitions may vary, HRQoL has been acknowledged as a multidimensional construct reflecting an individual’s quality of life by assessing physical, mental, emotional, and social functioning^9^. Several findings^10–12^ suggest that HRQoL may predict survival in various community settings and could be used to assess prognosis among PLWH in conjunction with demographic and clinical data^13^. Even though ART effectiveness has changed the current condition of PLWH, stigma is still present. In 1963 Goffman^14^ defined stigma as ‘an attribute which is deeply discrediting’. Stigmatized people are seen as deviant from normality, thus leading to be treated with less respect^15^. HIV stigma includes discrimination, social ostracism, violence and job loss^16,17^. Furthermore stigma and the fear of HIV positivity related to it negatively impact the the “Treat-all” HIV care^18^ and it has been associated with a lower ART adherence^19^, while internalised HIV-related stigma^20^ may play a critical role in treatment-related behaviours^21^ by leading to maladaptive coping strategies^22^ Neverthelss, scarce HRQoL has been associated with suboptimal HIV treatment outcomes, including inadequate engagement in care and adherence to ART and increased mortality, as well as poor mental health in both high-income and low- and middle-income countries.

Coping refer to the capability to reframe a stressfull event in your own life^23^. According to Carver, ther are maladaptive or adaptive coping strategies such as behavioral and/or mental disengagement and rumination or active approach and seeking social support. Among PLWH, coping strategies are crucial to overall health and adaptive coping was negatively correlated with enacted and anticipated stigma^24^. As recently suggested^25^, Patient-Reported Outcomes (PROs) should be considered as crucial in PLWH monitoring and become a standard part of mandatory outcome measures in both clinical daily practice and trials. Moreover, as for HIV-related stigma and HRQoL, PLWH can provide unique key-elements to identify their own unmet needs and vulnerabilities^26^.

The World Health Organisation (WHO) endorsed narrative research to complement the HRQoL-related findings of clinical and observational studies, to inform the refinement of survey tools and the introduction and implementation of new policies^27^. As described in previous projects^28,29^, Narrative Medicine (NM) deals with illness narratives^30^ to pursue the integration of the *disease*-centred biomedical sphere with the *illness*- and *sickness*-centred ones, concerning the individual and social experience of a condition, respectively^31^. In research, NM helps identify feasible interventions and implement the care pathway by integrating the perspectives of all the actors involved^32^; its findings have been increasingly used to improve the quality of care.

### Aim

This research article presents findings from narratives collected among participants in DIAMANTE (Italian Retrospective and Prospective observation of antiretroviral treatment in patients taking Darunavir/cobicistat plus eMtricitabine and tenofovir AlafeNamide fumarate), a non-interventional study which aimed to (a) prospectively describe the effectiveness of D/C/F/TAF treatment of HIV-1 in clinical practice and (b) collect retrospective data on previous ART treatments and establish their influence on the current ART effectiveness. The narratives collection included in the DIAMANTE study design aimed to assess how people live with from the HIV-positivity diagnosis to ART beginning, following its evolution over time. Although other observational studies and comparative trials applied PROs to different aspects of HIV clinical management, different ARTs efficacy and HRQoL assessment^33,34^, to the best of our knowledge, DIAMANTE was the first non-interventional study integrating narratives as PROs into clinical research on HIV in Italy.

## Methods

**Figure 1:**
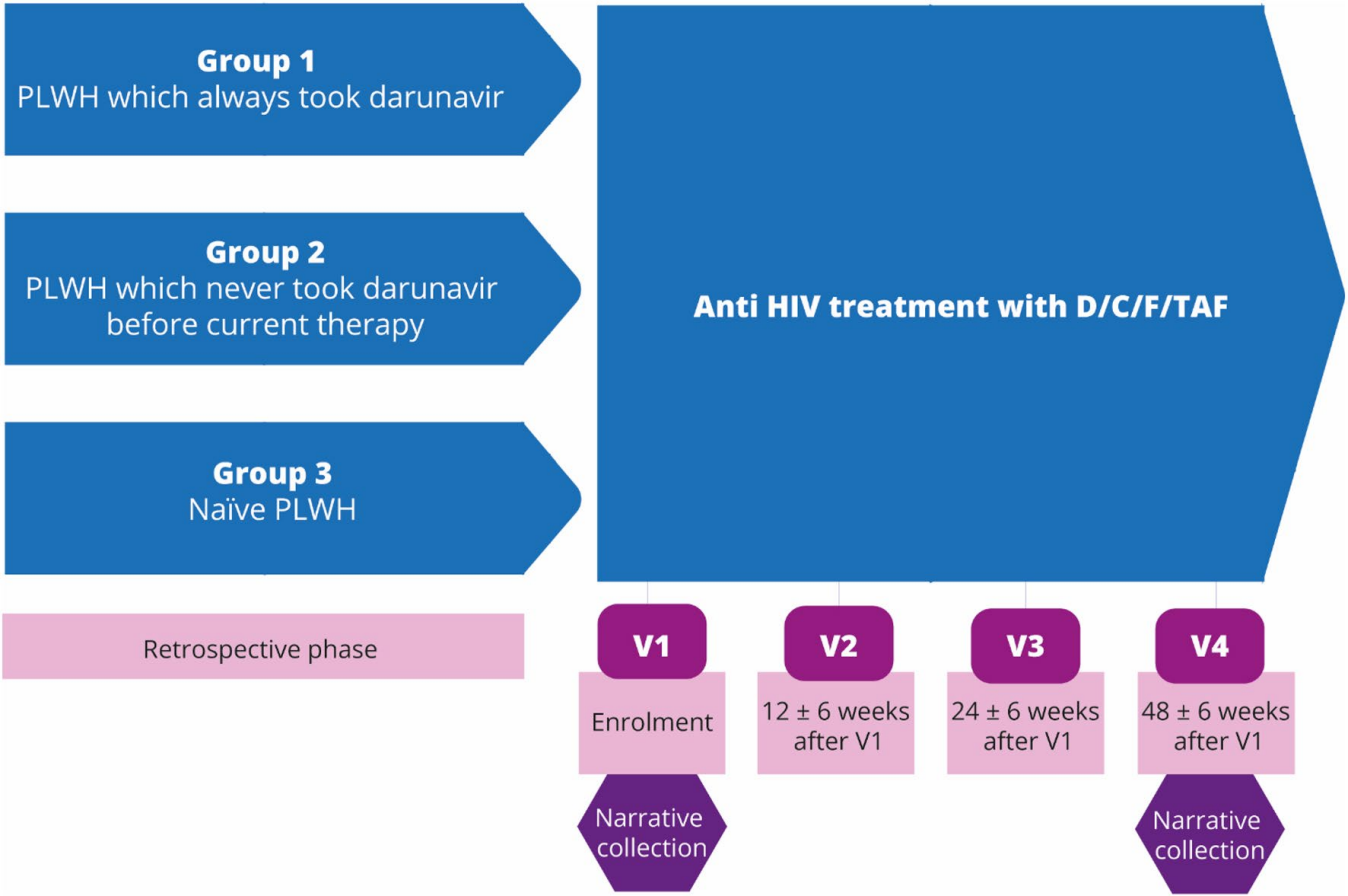
Study design and narrative collection time point.

The DIAMANTE study ran from June 2018 to September 2020 and involved 18 hospital-based centres for HIV care across Italy. Main inclusion criteria in the DIAMANTE study included patients aged ≥ 18, confirmed diagnosis of HIV-1 infection, the ability to sign an Informed Consent Form allowing data collection, D/C/F/TAF therapy started as per Summary of Product Characteristics (SmPCs) since at least one month before enrolment. Two-hundreds and forty-three PLWH were enrolled in the study. Participation in the narrative collection was voluntary and provided at enrolment (V1) and fourth visit (V4) 48 ± 6 weeks after V1 (Fig. 1). Treating clinicians underwent a webinar conducted by ISTUD researchers to be informed on the narratives collection’s purposes and design to better engage enrolled patients to share their written experiences.

### Data collection

Clinical data were collected as per clinical practice by the Principal Investigators and reported in the dedicated electronic-Case Report Form (eCRF); these data are not purpose of this publication. The narrative collection design followed a mixed-methods approach^35^. Both V1 and V4 narratives consisted of an illness plot^36^, and at V1 a quantitative questionnaire aimed at gathering sociodemographic data not included in the eCRF was added. The illness plots were jointly created by three ISTUD researchers aimed to chronologically guide the narrative to identify evolutions over time and characterised by evocative and open words to encourage individual expression^37^. The V1 questionnaire and illness plot (Supplementary file 1) did not coincided with those at V4 (Supplementary file 2), specifically designed to gather the participants’ experience after 48 ± 6 weeks after enrolment.

Treating clinicians handed PLWH the paper illness plots; patients could complete them at the centre or home, returning them at the next visit. PLWH narratives were collected by treating clinicians and sent to ISTUD researchers by mail. Researchers were blinded to preserve anonymity; V1 and respective V4 narratives were identifiable by a code.

### Ethical considerations

The narratives collection was performed according to the Declaration of Helsinki. Before their involvement, patients provided written informed consent, in addition to the DIAMANTE study’s informed consent, after being briefed by clinicians on narrative collection’s purposes and confidential data handling procedures, according to the Italian Law 196/2003^38^ and the European Union General Data Protection Regulation 2016/679^39^. The narratives collection was approved by each centre’s Ethical Committee. Furthermore, the unwillingness to participate to narrative collection did not prevent the patients’ enrolment in the DIAMANTE study.

### Analysis

Quantitative responses related to sociodemographic data were analysed through descriptive statistics through excel software. Narratives were professionally transcribed and were entered into Nvivo software to identify word recurrences and most common expressions to obtain clusters not predictable a priori. The illness plots were analysed by three ISTUD researchers with different academic backgrounds—anthropology, biotechnology, and epidemiology –, large experience in narrative research and NM. Ten V1 and their correspondent V4 narratives were collectively coded to assess consistency across researchers; afterwards, each narrative was coded separately and then reviewed within monthly peer debriefings which helped in reaching a shared data understanding and enhancing reflexivity among research team.

Researchers employed open interpretive coding to analyse identified topics and retrospectively applied Kleinman’s classification^31^ to identify *disease*-, *illness*-, and *sickness*-related aspects in narratives, respectively concerning the biomedical description of the condition, its personal experience, and its social perception; HIV metaphors used by PLWH in V1 narratives were analysed to trace spontaneous meaning associations created through daily language. Coping was assessed based on coping factor inventory by Carver^23^ and Launer’s narrative classifications^40^. The analyses of the narratives were reported both with the frequencies and extracts from the parallel charts translated into English.

After topics classification researchers evaluated how these identified elements influence coping in both V1 and V4 narratives. Statistical significance was assessed through the chi-square analysis^41^ to understand factors influencing coping strategies and McNemar analysis^42^, or McNemar-Bowker analysis^43^ to evaluate the evolution of the identified topics between V1 and V4. These analyses were performed through excel spreadsheets.

### Ethical approval

The protocol of the DIAMANTE study and amendments were approved by the ethics committee of the coordinating (Ethics Committee of The National Institute For Infectious Diseases "Lazzaro Spallanzani" IRCCS) and the participating centres. All patients provided written consent. The study is registered in the ClinicalTrials.gov database (NCT03577470).

### Consent to participate

Patients provided written informed consent after being briefed by clinicians on purposes and confidential data handling procedures.

## Results

Among the 243 patients enrolled in the DIAMANTE study, 114 (47%) completed both V1 and V4 narratives. Eighty-five percent were male; patients have been living with HIV for 2.66 years ± 2.75 on average. Table 1 summarises participants’ sociodemographic and anamnestic data.

**Table 1:** Sociodemographic and anamnestic data of participants.

	Participants to narratives collection (N = 114)
Sex	
Men	95 (85%)
Nationality	
Italian	105 (92%)
Other	9 (8%)
Age, years	41.5 ± 11.5 (18–68)
Civil status	
Married or with a partner	34 (30%)
Single	60 (54%)
Divorced or widow	18 (16%)
With children	33 (29%)
Education level	
Elementary or middle school diploma	30 (26%)
High school diploma	48 (42%)
Bachelor’s degree or higher education	36 (32%)
Work situation	
Self-employed worker	23 (20%)
Employee	66 (59%)
Not working	15 (13%)
Student	4 (4%)
Retired	4 (4%)
Living with HIV, years	2.66 ± 2.75 (0–19)
Previous therapies (at study entry)	
Having always been treated with DRV since the start of ARV treatment as naïve	42 (37%)
Not having been treated with DRV before starting of D/C/F/TAF	16 (14%)
Antiretroviral Naïve (any viral load)	56 (49%)
Way of infection	
Blood contact	1 (1%)
Men who have sex with men (MSM)	65 (57%)
Heterosexual	41 (36%)
Intravenous drug use	0
Unknown	7 (6%)
HIV RNA (copies/ml) at V4	
< 50	102 (89%)
> 50	12 (11%)

Data are presented as n (%) or mean ± SD (range).

Patients chose their current reference centre based on territorial proximity (24%), high excellence reputation (26%), or recommended by other healthcare professionals (35%); 15% of patients did not specify the reason of their choice for the reference centre. Overall, patients appreciated the opportunity to share their experience in writing (86% at V1, 89% at V4); in particular, 44% defined this experience as satisfying and liberatory at V4.

Narratives were analysed to evaluate if patients accepted HIV-infection diagnosis while developing adaptive coping strategies. Separate analyses of V1 and V4 narratives showed no major differences in coping, which remained stable between 51 and 59%, with no statistically significant differences considering treatment groups. Therefore, findings from the narratives have been correlated with coping to understand elements promoting or inhibiting positive acceptance of HIV-positivity diagnosis.

The presentation of LWH-related findings will focus on those dimensions showing statistical significance (*p* value) with coping strategies, ordered as follows: (a) experiences analysed through narrative classifications and metaphors; (b) previous HIV knowledge before diagnosis and emotions experienced throughout the care pathway; (c) the decision to share or not HIV-positive status within relatives and friends; (d) HIV-positivity experience within work and activity sphere.

### LWH through narrative classifications and metaphors

Ninety-two percent of V1 and 89% of V4 narratives highlighted *illness*-related issues and terminology (Table 2); at V4, a major focus emerged on issues linked to the clinical and technical *disease* aspects. The social dimension of *sickness* emerged in both V1 and V4 narratives (64% and 49%, respectively) and referred to the stigma still present within the society. At V4, patients mentioning *sickness*-related aspects (46%, *p* = 0.027) were those coping less.

**Table 2:** *Disease*-, *illness*-, and *sickness*-related aspects: quotes from narratives.

Disease	—*Today, living with HIV is much easier. The change in therapy and the reduction of tablets has benefited psychologically* —*I started to learn about the therapy and the experiences of those who had gone through it before me. The treatment I was given was new and therefore worried me*
Illness	—*Like any other person, or rather like a normal person, and even if I know that the disease is there, it does not mean that I cannot live it to the fullest as I have always done and be more careful in the things I do* —*As if time were short. As if everything was vital. Inside myself, however, I try to hide my insecurities which make me put up concrete walls. I feel stronger than before. But I never talk to anyone about certain things, making me feel insecure*
Sickness	—*I do not talk about it. Only two people know about it. I don't think my friends would understand either. I am sure they would change their attitude. I would be afraid of being judged* —*Because of the well-known mistrust of people with this disease, it is impossible to address this topic*

HIV metaphors were clustered into three groups: (a) 41% recalled a malignant nature or a constant presence (—*A black cloud overhead*); (b) 38% referred to monstrous or stigma-related figures (—*A stain on a perfect painting that will never be forgiven*); (c) 21% indicated HIV as a mate, a journey, or a battle to be faced (—*A second chance at life*).

### LWH from HIV knowledge before diagnosis to current emotions

58% of patients chose not to narrate their life before the HIV-positivity diagnosis, while 9% reported a problematic past, often made of loneliness and abuse. Conversely, 33% mentioned positive feelings related to their life before the diagnosis of HIV-positivity. At V4, patients who described a pleasant past showed most success in evolving from non-acceptance to coping (72% at V4 vs 54% at V1, *p* = 0.045); in contrast, patients who chose not to narrate their past (49%) were the least successful in coping.

Forty-six percent of patients showed previous poor HIV knowledge; only 19% reported knowing other PLWH and for 29% HIV still represented death. Forty-three percent of patients found out their HIV-positivity condition through purposely requested screening tests, and 15% through tests addressed to work-related screening or other special situation (e.g., pregnancy); however, 42% discovered the condition while facing HIV-related complications, e.g., fever, asthenia, or pneumonia. Analyses showed that the way PLWH found out HIV-positivity did not influence coping both at V1 (*p* = 0.6445) and V4 (*p* = 0.8508).

**Figure 2:**
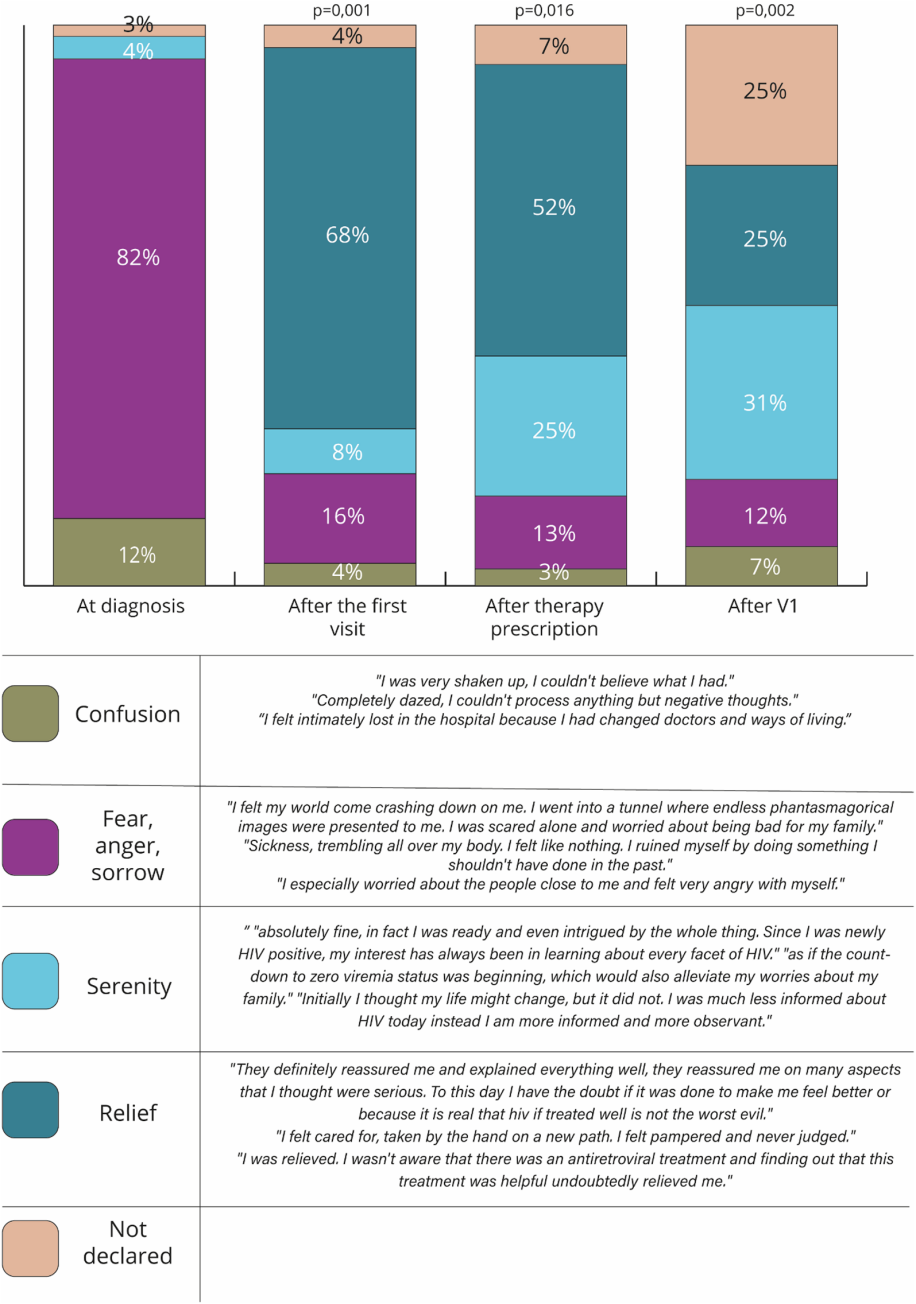
PLWH emotions at different timepoint as described in the 114 narratives collected.

In narratives, patients reported the emotions they felt throughout the care pathway (Fig. 2); at HIV-positivity diagnosis, 82% felt fear, anger, and sorrow, followed by confusion (12%), while at V1 68% felt relief since clinicians reassured them, explaining the condition and therapies to control it. The very beginning of therapies corresponded to further relief (52%) and serenity (25%). At V4, patients recalled the emotions felt at V1, dwelling on relief (25%) and serenity (31%); however, participants experiencing confusion remained noticeable since they coincided with patients coping less (7%, *p* = 0.002).

At V1, 30% of patients reported they were keeping their HIV-positivity private with anyone (Table 3); this percentage drops to 16% at V4. However, at V1 only 3% reported that they openly talked about their condition; 67% decided to talk about HIV-positivity status only with their own family or reliable friends. At V4, patients who openly talked (6%) about HIV-positivity coped more (100%, *p* = 0.012); conversely, PLWH who maintained the “secret” coped less with their condition (35%, *p* = 0.012).

**Table 3:** Sharing the HIV-positive status with other: quotes from narratives.

Maintaining privacy with all	—*I decided not to say anything to my family members, or anyone, and go through this alone*
Everyone knows	—*I immediately wanted to tell everyone. Relatives and strangers, to be sincere from the beginning, except at work, where I said nothing for fear that it would compromise my activities*
Only family and trustworthy friends	—*My parents and best friends know about my condition, and they have never excluded me, which is very important. For the past two months, I have been in a relationship with someone who has peacefully accepted my condition, and we live our story in serenity* —*I decided to call family members right away. My cousin was the first to know… So many tears we shed…*

### Relationships with relatives and friends and coping with HIV-positivity

**Figure 3:**
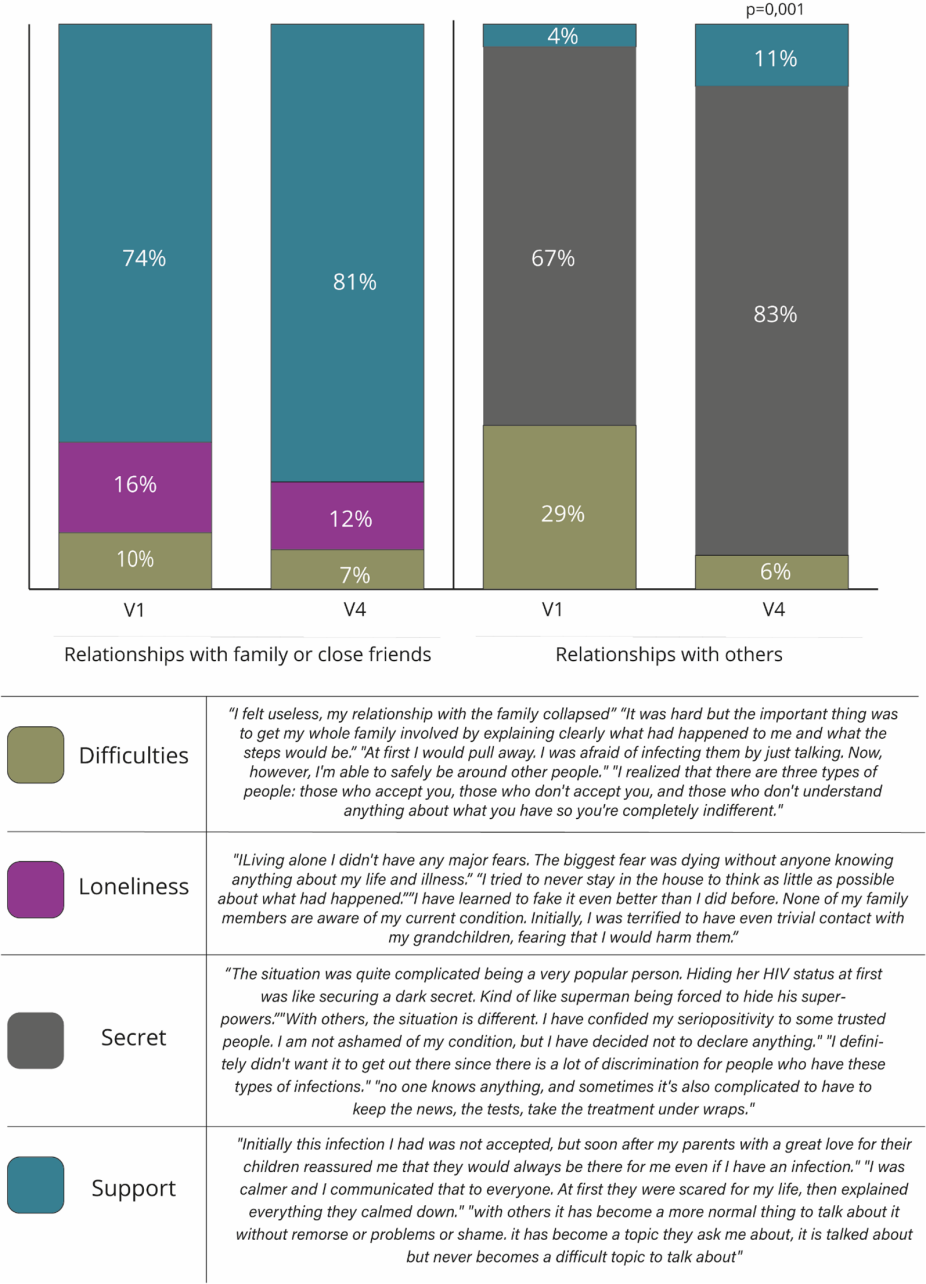
Relationship evolution from V1 to V4 as stated in the 114 PLWH narratives.

Seventy-four percent and 81% of patients respectively at V1 and V4 reported to feel supported and understood by their relatives (Fig. 3), developing more capability to cope with HIV-positive status (from 56% at V1 to 69% at V4, *p* = 0.045)—this percentage dropped to zero for patients facing difficulties in the familial context. As for relationships external to the family context, the percentage of patients who preferred to completely isolate themselves decreased from 29% at V1 to 6% at V4; nonetheless, at V4, 83% of patients kept their HIV-positive status secret with others. Positively, at V4, percentage of patients succeeding in developing supportive relationships increased from 4 to 11%, these patients were also more successful in coping with the condition (92% at V4, *p* = 0.001).

At V1, fear of HIV impact on sexual life represented a spontaneously emerged issue in 23 of 114 patients; at V4, 88% of these patients reported to be no longer afraid to infect sexual partners, and this helped them to accept their HIV-positive status (*p* = 0.023).

At V4, 72% of patients described their relationship with clinicians as excellent (Table 4); conversely, 6% complained difficulties since the hospital organisation did not guarantee them to be visited by the same clinician; moreover, PLWH who expressed difficult relationship with clinicians coped less (17%, *p* = 0.040). Also, 26 of 114 patients spontaneously stated their pleasure in having participated in this study since they felt better followed and more informed on their condition.

**Table 4:** The relationship with treating clinicians: quotes from narratives.

Good or excellent relationship	—*Definitely great. I can relate and ask many questions with answers that reassure me* —*It is very good. It creates understanding and mutual trust, which is essential in a relationship* —*Very good. He always informs me about my physical condition, and we also chat occasionally about more and less. He is an excellent doctor, attentive and thorough* —*Satisfactory, and I believe it is fundamental to the empathy created between doctor and patient. This is worth at least 20% of the success; the pill does the rest*
Challenging hospital organisation	—*I do not have a specific idea about this. I have often changed doctors because of the hospital’s organisation (or disorganisation)* —*The doctors are competent and helpful even though the structure is upsetting due to internal reorganisation […]. All this has been passed on to the patients who have lost the famous one-to-one relationship that made the hospital unique and special, which was also why I chose other healthcare facilities*
Participation in the study	—*Within this clinical trial, I felt followed and cared for* —*I was happy to be included in this study. This is because I felt even more followed. In short, it gave me the feeling or instead reinforced that I was being followed even more closely* —*Knowing about the possibility of entering a “protocol” dynamic […] I saw it as a “taking care of me”—not in a generalised sense, but as a free request to be able to follow side by side my treatment pathway with targeted drugs and treatments, which would then give the expected results so that, from my successful outcome, for example, a “good pathway-approach” could come out for other people in my same situation. This is certainly not a “fast track” but rather a moment of responsibility for me, of full availability so that the succession of treatment is a litmus test—no longer extraordinary but ordinary for others. So, armed, but also serene, in accepting this path, I let myself be “taken care of”—such seems the appropriate term […] because it is taking care, having a more “targeted” index of attention towards me. And I perceived this individually from each person in charge of my care*

### HIV-positive status at workplace and in daily activities

At V4, 78% of patients who narrated their working situation (n = 87) highlighted to maintain the privacy on their condition even at workplace (Table 5). At V1, 18% of these 87 patients faced dismissal or demotion due to HIV-positivity or numerous HIV-related sick leaves; at V4, only 1% of these patients still reported these issues in their narratives. At V4, 7% of these patients stated to have shared their condition at the workplace and found comprehension by their colleagues. Moreover, comparing coping with the work situation reported within the questionnaire, employees showed to cope more than self-employed or not working patients.

**Table 5:** HIV-positivity experiences at workplace: quotes from narratives.

Maintain secrecy	—*I am always discreet and try to never mention it to anyone* —*Nothing has changed, no one knows anything, and I continue to work the same way*
Dismissal or demotion due to HIV-positivity	—*I have had a little agreed-upon demotion—I do not do overnight on-call, and I do not have patient contact anymore. Still, I do take care of other essential things. So, everything is going well* —*Unfortunately, I was fired for HIV, but it was the impetus to create something of my own and new*
HIV-positivity sharing with colleagues	—*They know about me, and after some time, nothing has changed. I don't see how it would be if I changed job. I feel lucky*

Patients reported that they had no problems in performing activities, except for a very early period related to HIV complications (83% at V4); at V4, 10% of 86 patients referring on activities stated to have started doing even more activities than before. Between V1 and V4, 22 (19%) patients referred that they decided to improve their care for physical wellness after HIV-positivity diagnosis. However, 7% persisted in isolating themselves at V4 because of the psychological burden due to HIV-positivity and corresponded to patients who did not accept the condition and therefore could not cope (*p* = 0.013).

## Discussion

Collecting narratives as PROs within the DIAMANTE study allowed to highlight new elements of LWH beyond validated questionnaires and clinical data.

The analysis of narratives showed that the previous therapeutic history and therapeutic failure assessed according to the parameters of this study do not influence coping; moreover, in contrast to what has been reported in literature^21,44^, neither the viral load itself nor the time elapsed since the knowledge of the disease seems to impact the coping process. In fact, no difference was observed between naïve and antiretroviral -experienced patients. On the contrary, being on treatment plays a crucial role in reassuring patients about their health status, their risk of death and the risk of being no longer infectious for their partners. The relationship with the treating clinician is perceived to be pivotal since the first visit, when patients mostly need to be supported through extensive information about treatment efficacy, tolerability of medications, durability of response and return to a normal life. This finding is in line with other studies highlighting the importance of the patient-treating physician as a crucial predictor of PLWH acceptance of and adherence to ART, also outside the Italian context^45,46^. The sense of relief experienced during the visits helps PLWH coping with the stressful emotions following the diagnosis. By contrast, difficult relationships with treating clinicians or a challenging therapeutic pathway make coping more complex.

In addition, while the current clinical features do not seem to influence coping, the acceptance of new status (i.e., being HIV infected) seems to directly influence how antiviral treatments are perceived. The narratives suggest that some participants perceived HIV medications as a daily reminder about their illness and this may ultimately affect treatment adherence, as reported in literature^47^.

From the analysis of narratives, an increase in HIV-positivity acceptance emerges when PLWH establish supportive relationships with family members, friends, or colleagues: these relationships, as well as avoiding isolation or secrecy because of their status, reduce stigma and help the return to a “normal” life. The opportunity to narrate and share their HIV-related experience emerges as the most helpful strategy for PLWH to cope with the disease. Indeed, self-disclosure has been proven to be beneficial for HIV prevention and adherence, and several studies tried to define a model for disclosure^48–50^

Notably, very few participants reported to have directly experienced stigma or discrimination, mainly referring to the workplace, due to the disbelief that the HIV infection might be spread in that setting, during daily activities (i.e., chefs, fireman). PLWH didn’t shared invisible stigma examples.

Narratives and metaphors show that the stigma perceived by society is internalised by patients through shame, guilt, and embarrassment and can be observed especially in V1 narratives, in those participants who decided not to narrate their story before HIV infection diagnosis and rethought their experience with a sense of guilt. Internalised stigma, however, seems to decrease over time through supportive relationships and sharing their experience of LWH, as shown by V4 narratives; this element may integrate other findings showing a higher internalised stigma score when care engagement for PLWH is challenging^51^.

The writing experience was perceived positively by participants and, in some cases, helped them reflect on the experience and understand personal emotional growth during the last year. Furthermore, through writing patients felt listened, welcomed, and supported in their journey with HIV.

DIAMANTE is the first observational study integrating narratives as PROs and clinical data in Italy: thus, it is line with those studies suggesting that implementing PROs in clinical practice should represent a key-aspect of LWH strategic management and patient-centred medicine^25^.

One limitation of this narrative collection is that only the patients selected to participate in the study are represent and, among them, only the ones who felt comfortable with the process of writing. In fact, only 47% of patients enrolled in the DIAMANTE study completed both V1 and V4 narratives. Since participation to the narrative research was optional, we have no data on why participants decided not to write their stories Nevertheless, being able to fluently write in Italian, and time constrain are the main reasons reported by principal investigators.

## Conclusion

This narratives collection aimed to assess HRQoL since the HIV infection diagnosis to ART beginning, following its evolution over time. Coping with the HIV infection is a complex experience, requires time and is positively affected by the relation with the treating physicians. The first medical visit may strongly reassure patients and being on therapy may help the coping process. Using narratives as PROs provided new insights on living with HIV in Italy and PLWH coping elements to overcome internalised stigma, fostering clinical knowledge and PLWH awareness of their condition.

### Supplementary Information

Supplementary Information 1.

Supplementary Information 2.

## Data Availability

All results are available upon request to the corresponding author and upon the respect of privacy requirements. Narratives are available in Italian.

## Abbreviations

WHO: World Health Organisation
ART: Antiretroviral therapy
PLWH: People living with HIV
HRQoL: Health-related quality of life
NM: Narrative medicine
LWH: Living with HIV
PROs: Patient-reported outcomes
SmPCs: Summary of product characteristics
