# Supplementary material for: Investigating coping and stigma in people living with HIV through narrative medicine in the Italian multicentre non-interventional study DIAMANTE
Source: Sci Rep. 2023 Oct 17;13:17624. doi: 10.1038/s41598-023-44768-2 (PMC10582167; doi:10.1038/s41598-023-44768-2)
Supplement: Supplementary file 1 — Supplementary Information 1. [file 41598_2023_44768_MOESM1_ESM.docx]

# Supplementary file 1

We kindly invite you to tell your story from the moment a change occurred in your life, when the first symptoms appeared to the present day. Feel free to write instinctively, regardless of the form and length of the story. Any episodes that you consider significant or narrative that you would like to include in the text will be welcome.

**Before the discovery of HIV**

Before the discovery of HIV, I felt...

I thought at the time that HIV...

The people in my life...

At work...

And in my spare time...

**The discovery of HIV**

The way I found out I was HIV-positive was…

The moment I received the results of the HIV test, I felt…

I decided to (to say or not to say, to do or not to do)…

Waiting for my first visit with an infectious diseases docto …

During the first visit I felt…

While immediately after the first visit with an infectivologist…

At home…

With others…

And I could…

While I could not…

So I felt…

And I wanted…

**HIV care**

When I was informed of the need to change/start antiretroviral therapy, I felt…

And I thought…

Today living with HIV is…

I feel…

Today I can…

My relationship with my doctor is…

Treatment to date…

At home…

At work…

And in my free time…

The people in my life…

Thinking about the journey so far…

When I think about tomorrow…

And I would like to…

Thank you for your time, energy and thought.

We ask you one last question:

How did it feel to be able to recount your experience?
