# Supplementary material for: Investigating coping and stigma in people living with HIV through narrative medicine in the Italian multicentre non-interventional study DIAMANTE
Source: Sci Rep. 2023 Oct 17;13:17624. doi: 10.1038/s41598-023-44768-2 (PMC10582167; doi:10.1038/s41598-023-44768-2)
Supplement: Supplementary file 2 — Supplementary Information 2. [file 41598_2023_44768_MOESM2_ESM.docx]

# Supplementary file 1

We kindly invite you to tell your story from the moment a change occurred in your life, when the first symptoms appeared to the present day. Feel free to write instinctively, regardless of the form and length of the story. Any episodes that you consider significant or narrative that you would like to include in the text will be welcome.

**After the first visit of the DIAMANTE study**

Immediately after the first visit of the DIAMANTE study…

At home…

With others…

And I could…

While I could not…

So I felt…

And I wanted…

**Today**

Living with HIV today is…

I feel…

Today I can…

HIV care…

My relationship with my doctor is…

At home…

At work…

And in my free time…

The people in my life…

Thinking about the journey so far…

When I think about tomorrow…

And I would like to…

Thank you for your time, energy and thought. We would like to ask you one last question:

How did it feel to be able to recount your experience from the moment you joined the DIAMANTE study?
